# A nationwide retrospective study of the July effect on nonvariceal upper GI bleeding outcomes in teaching hospitals in the United States

**DOI:** 10.1186/s12876-026-05035-6

**Published:** 2026-06-30

**Authors:** Adedeji Adenusi, Olamide Asifat, Bright Nwatamole, Geraldine Nabeta, Chima Amadi, Joseph Atarere, Oluwatayo Awolumate, Eugene Annor

**Affiliations:** 1 Department of Internal Medicine, Interfaith Medical Center, Brooklyn, NY USA; 2 Obafemi Awolowo University, Ile-Ife, Osun State Nigeria; 3 Jiann-Ping Hsu College of Public Health, Georgia Southern University, Statesboro, GA USA; 4 Vassar Brothers Medical Center / Nuvance Health, Poughkeepsie, NY USA; 5 Department of Medicine, University of Connecticut Health Center, Farmington, CT USA; 6 Department of Medicine, Morehouse School of Medicine, Atlanta, GA USA; 7 Department of Medicine, MedStar Union Memorial Hospital, Baltimore, MD USA; 8 Department of Internal Medicine, Howard University Hospital, Washington, DC USA; 9 Department of Medicine, University of Illinois College of Medicine, Peoria, IL USA

**Keywords:** Upper gastrointestinal bleeding, Esophagogastroduodenoscopy, July effect, Mortality, Length of hospital stay

## Abstract

**Background:**

“July effect” also known as “July phenomenon” are adverse patient outcomes related to the changeover of medical residents in teaching hospitals in the United States at a particular time of the year. This study aims to explore this phenomenon on hospital outcomes among patients with nonvariceal upper gastrointestinal bleeding (UGIB).

**Methods:**

The National Inpatient Sample data from Jan 1, 2016, to Dec. 31, 2020, was used. Patients admitted in teaching hospitals between May and August were stratified patients into two groups; May & June; as (Non-July Effect), July & August as (July Effect). The study involved a comprehensive assessment and comparison of various clinical outcomes, including the likelihood of mortality, length of hospital stay, and the utilization and timing of esophagogastroduodenoscopy (EGD). These analyses were conducted while controlling factors such as socio-demographic variables, hospital characteristics, and associated comorbidities to ensure accurate and meaningful results.

**Results:**

A total of 53,710 patients had non-variceal UGIB. There is no significant difference in Mortality risk (aOR = 1.10, 95% CI: 0.86–1.40, *p* = 0.4406), Length of hospitalization (0.98, 0.91–1.05, *p* = 0.5410), EGD (1.04, 0.94–1.32, *p* = 0.4412), and Early EGD (0.97, 0.80–1.19, *p* = 0.7759) between the July effect and non-July effect groups.

**Conclusions:**

The analysis reveals no significant differences in mortality rates, length of hospitalization, or endoscopy and its timing among patients with UGIB during the July effect compared to those not affected by this phenomenon. Our study corroborates previous research on the July phenomenon while also exploring this phenomenon in upper gastrointestinal bleeding.

## Background

The “July effect” was first described in 1989, referring to the perceived impact of new trainees joining teaching hospitals in the academic year. While a study at the time found no significant increase in the total costs of care during this period [1], the concept has persisted in medical terminology. The “July phenomenon,” or “March effect,” or “August killing season,” or “Black Wednesday” (depending on the country), suggests that the influx of new residents and fellows leads to a rise in adverse events and medical errors [2]. This theory attributes adverse outcomes to limited clinical knowledge and experience among new trainees, compounded by the disruption of established care routines [3].

Upper gastrointestinal bleeding (UGIB) is one of the most common medical emergencies, carrying a mortality risk as high as 10% [4]. Nonvariceal upper gastrointestinal bleeding (NVUGIB), the most common subtype, is primarily caused by peptic ulcers, with other etiologies including esophagitis, Dieulafoy’s lesions, angiodysplasia, neoplasms, Mallory-Weiss tears, and gastric antral vascular ectasia (GAVE) [5].

Esophagogastroduodenoscopy (EGD) is the gold standard for diagnosing and managing UGIB, as it allows for direct therapeutic interventions, identifying up to 90% of bleeding sites. American College of Gastroenterology (ACG) recommends performing the procedure within 24 h to maximize clinical and economic outcomes [6]. This significantly impacts patient outcomes, particularly for high-risk individuals, who may benefit from prompt and coordinated interventions [7]. Early endoscopy has been shown to reduce hospital length of stay and costs for patients with NVUGIB, regardless of admission day [8]. Despite the established benefits of endoscopy in achieving hemostasis and preventing complications, the impact of trainee inexperience on outcomes during the academic year’s early months remains underexplored.

Procedural complications such as perforation and bleeding occur at rates of 10 and 75 per 100,000, respectively, while the 30-day mortality rate following EGD is approximately 34 per 100,000 [9]. Evidence suggests that these complications may correlate with the academic year’s timing, as trainee transitions could contribute to adverse outcomes [10].

Mixed findings regarding the July effect on patient care, particularly for UGIB, highlight the need for further investigation. For instance, a study by Asotibe et al. demonstrated worse outcomes for UGIB patients in teaching hospitals, particularly from July to September, coinciding with the start of GI fellows’ training [11]. Similarly, Phillips and Baker et al. reported an increase in fatal medication errors in July, linked to teaching hospitals [10]. On the other hand, a meta-analysis by Zogg et al. found no evidence of the July effect for major adverse outcomes, suggesting that factors such as heightened supervision and better team coordination may mitigate risks [2]. Additionally, a study by Gangu et al. observed no significant differences in procedural complications for EGDs performed in July through September, though prolonged hospital stays were noted, possibly influenced by new trainees across specialties [9].

This study aims to explore and compare the July effect on hospital outcomes and endoscopic treatment among patients with UGIB. By examining this phenomenon, we seek to provide insights into the effects of the July transition of residents and fellows on clinical care and identify areas for improvement in patient safety and trainee education.

## Methods

### Data source and design

We reviewed the National Inpatient Sample (NIS) data from Jan 1, 2016, to Dec. 31, 2020, to examine the effect of time of year. NIS is a Healthcare Cost and Utilization Project (HCUP) data, which is the largest publicly available inpatient healthcare database developed by Agency for Healthcare Research and Quality (AHRQ). It provides estimates of inpatient utilization, access, cost, quality, and outcomes of about 35 million hospitalizations in the U.S. This database was reported using the International Classification of Diseases, Tenth Revision, Clinical Modification/Procedure Coding System (ICD-10-CM/ PCS) for the period of study.

This cross-sectional study was conducted and reported in accordance with the STROBE (Strengthening the Reporting of Observational Studies in Epidemiology) guidelines to ensure transparent reporting of observational research. The Inclusion criteria were patients admitted from May through August over the 5-year period, while the exclusion criteria were patients admitted in non-teaching hospitals, admitted before May and after August, and patients diagnosed with variceal upper gastrointestinal bleeding. The study population was then stratified into two groups; those admitted in May & June (Non-July Effect) and those admitted in July & August (July Effect) and with primary diagnosis of UGIB. The dependent or outcome variables are mortality, endoscopy, early endoscopy and length of hospitalization. The independent or exposure variables are July effect, socio-demographic, hospital characteristics, total hospital Cost, associated comorbidities (respiratory failure, cardiac arrest, chronic kidney disease, hypovolemic shock). A weighed descriptive analysis of study characteristics, using the variable ‘DISCWT’ (Discharge Weight) to reduce sampling bias in the survey was done. This continuous variable; length of stay was recategorized to categorical variables (≤ 3 days vs. >3 days) to fit into the logistic regression analysis. Missing data was assessed for all study variables. Records with missing values for key outcome or exposure variables were excluded from the analysis.

Chi-square test was performed to compare categorical variables, bivariate and multivariate logistic regressions to identify and compare the primary outcomes; in-hospital mortality, longer hospital stay, and EGD and Early-EGD (< 24 h) among the two groups. Survey weights were applied in the regression models, generating nationally representative estimates. All analyses were conducted with statistical software SAS version 9.4 (SAS Institute Inc., Cary, NC, USA) and the level of significance was set at p-value ≤ 0.05.

## Results

### Descriptive analysis

A total of 53,710 (weighted frequency) patients were admitted from May through August with a diagnosis of upper gastrointestinal bleeding (UGIB) as shown in Table 1. Among these, less than 5% deaths during hospitalization, approximately 32% had a length of stay exceeding three days, and 16% underwent EGD of which 25% of them had it < 24 h from admission. The study population was evenly distributed between the July effect group (patients admitted in July–August: 50.61%) and the non-July effect group (May–June: 49.39%).

**Table 1:** Study characteristics by July and Non-July effect in teaching hospitals (N-53710)

Study characteristics	July Effect	Non-July Effect	*P*-values
Age			0.6945
18–34 years	1350 (3.44%)	1265 (3.30%)	
35–44 years	2190 (5.58%)	1995 (5.21%)	
45–54 years	3520 (8.97%)	3445 (8.99%)	
55–64 years	7290 (18.57%)	6935 (18.10%)	
≥ 65 years	24,905 (63.44%)	24,675 (64.40%)	
Esophago-duodenoscopy (EGD)			0.9640
No	33,190 (84.55%)	32,385 (84.52%)	
Yes	6065 (15.45%)	5930 (15.48%)	
Time of EGD			0.7628
< 24 h	1565 (25.80%)	1500 (25.30%)	
≥ 24 h	4500 (74.20%)	4430 (74.70%)	
Insurance			0.7457
Medicaid	4780 (12.18%)	4425 (11.55%)	
Medicare	25,710 (65.45%)	25,205 (65.78%)	
Other	1050 (2.67%)	1060 (2.77%)	
Private	6125 (15.60%)	5990 (15.63%)	
Selfpay	1590 (4.05%)	1635 (4.27%)	
Teaching status of hospital			0.6979
Urban teaching	27,195 (100%)	26,515 (100%)	
Median household income			0.6979
1st Quartile	12,475 (31.78%)	12,500 (32.62%)	
2nd Quartile	11,120 (28.33%)	10,785 (28.15%)	
3rd Quartile	8580 (21.86%)	8190 (21.38%)	
4th Quartile	7080 (18.04%)	6840 (17.85%)	
Sex			0.8906
Male	20,345 (51.83%)	19,900 (51.94%)	
Female	18,910 (48.17%)	18,415 (48.06%)	
Race/ethnicity			0.2816
White	27,935 (71.16%)	27,335 (71.34%)	
Black	6105 (15.55%)	6230 (16.26%)	
Hispanic	4135 (10.53%)	3755 (9.80%)	
Asian/Pacific Islander	1080 (2.75%)	995 (2.60%)	
Respiratory failure			0.6558
No	38,165 (97.22%)	37,205 (97.10%)	
Yes	1090 (2.78%)	1110 (2.90%)	
Hospital Bed size			0.0575
Small	9525 (24.26%)	8970 (23.41%)	
Medium	11,650 (29.68%)	11,990 (31.29%)	
Large	18,080 (46.06%)	17,355 (45.30%)	
Hospital region			0.3515
Northwest	7800 (19.87%)	7495 (19.56%)	
Midwest	8205 (20.90%)	7655 (19.98%)	
South	16,250 (41.40%)	16,275 (4.48%)	
West	7000 (17.83%)	6890 (17.98%)	
Shock			0.4309
No	38,745 (98.70%)	37,870 (98.84%)	
Yes	510 (1.16%)	445 (1.16%)	
Cardiac arrest			0.2741
No	39,195 (99.85%)	38,280 (99.91%)	
Yes	60 (0.15%)	35 (0.09%)	
AKI			0.8830
No	34,975 (89.10%)	34,165 (89.17%)	
Yes	4280 (10.90%)	4150 (10.83%)	
Mortality			0.1505
No	38,085 (97.02%)	37,320 (97.40%)	
Yes	1170 (2.98%)	995 (2.60%)	
Length of stay			0.9494
≤ 3 days	27,020 (68.83%)	26,355 (68.76%)	
> 3 days	12,235 (31.71%)	11,960 (31.21%)	
Total Hospital charge			0.6486
1st Quartile	5455 (13.90%)	5180 (13.52%)	
2nd Quartile	10,920 (27.82%)	10,800 (28.19%)	
3rd Quartile	13,940 (35.51%)	13,375 (34.91%)	
4th Quartile	8940 (22.77%)	8960 (23.36%)	

*AKI: Acute Kidney Injury; Non-July Effect (May & June); July Effect (July & August)

Most patients were White (71.25%) and male (51.88%), with the majority aged 65 years or older (63.92%). Admissions primarily occurred in large hospitals (45.68%) and were concentrated in the Southern region of the U.S. (41.92%). Regarding insurance status, Medicare was the most common payer (65.64%), followed by private insurance (15.62%) and Medicaid (11.87%).

In terms of socioeconomic characteristics, over 60% of patients were from the two lowest quartiles of median household income, suggesting a potential association between lower socioeconomic status and risk of UGIB hospitalization. Comorbidities and complications such as acute kidney injury (AKI), respiratory failure, shock, and cardiac arrest were relatively infrequent but significantly impacted outcomes. Notably, AKI was present in 10.87% of admissions, respiratory failure in 2.84%, shock in 1.23%, and cardiac arrest in 0.12%.

### Bivariate and multivariate logistic analyses of outcomes

The primary analysis showed no statistically significant differences between the July and non-July groups in the key outcomes assessed. The adjusted odds ratio (aOR) for mortality was 1.10 (95% CI: 0.86–1.40, *p* = 0.4406), and the unadjusted odds ratio was 1.03 (95% CI: 0.82–1.30, *p* = 0.7722), suggesting that resident turnover during July and August was not associated with increased inpatient mortality among patients with UGIB.

Similarly, for length of hospitalization, patients admitted during the July effect period showed no significant difference compared to those admitted in May or June. The adjusted odds ratio was 0.98 (95% CI: 0.91–1.05, *p* = 0.540), and the unadjusted odds ratio was 1.01 (95% CI: 0.94–1.09, *p* = 0.8203), indicating no meaningful association between the timing of resident transitions and prolonged hospitalization.

In evaluating other predictors of extended hospital stay, younger patients had significantly higher odds of prolonged hospitalization relative to older adults. For example, patients aged 18–34 had an aOR of 2.09 (95% CI: 1.74–2.50), and those aged 35–44 had an aOR of 1.81 (95% CI: 1.57–2.09), compared to the reference group aged ≥ 65. Self-pay patients also had increased odds of longer stays (aOR = 2.10, 95% CI: 1.72–2.57), highlighting potential disparities tied to socioeconomic status.

Regarding the likelihood of undergoing endoscopy (EGD), there were no significant differences between July and non-July groups (aOR = 1.04, 95% CI: 0.94–1.32, *p* = 0.4412). However, younger age groups were significantly less likely to receive EGD, particularly patients aged 18–34 (aOR = 0.52, 95% CI: 0.35–0.77). Hispanic patients had slightly lower odds of undergoing EGD (aOR = 0.82, 95% CI: 0.68–0.99), and both Medicaid (aOR = 0.75, 95% CI: 0.60–0.94) and self-pay patients (aOR = 0.62, 95% CI: 0.41–0.93) were less likely to receive endoscopy compared to those on Medicare.

The probability of early (< 24 h) EGD is not significantly affected when comparing differences between July and non-July groups (aOR = 0.97, 95% CI: 0.80–1.19, *p* = 0.7759). However, male was found to less likely get EGD < 24 h compared to females between July and non-July groups (aOR = 0.79, 95% CI: 0.64–0.99, *p* = 0.0370).

**Fig. 1:**
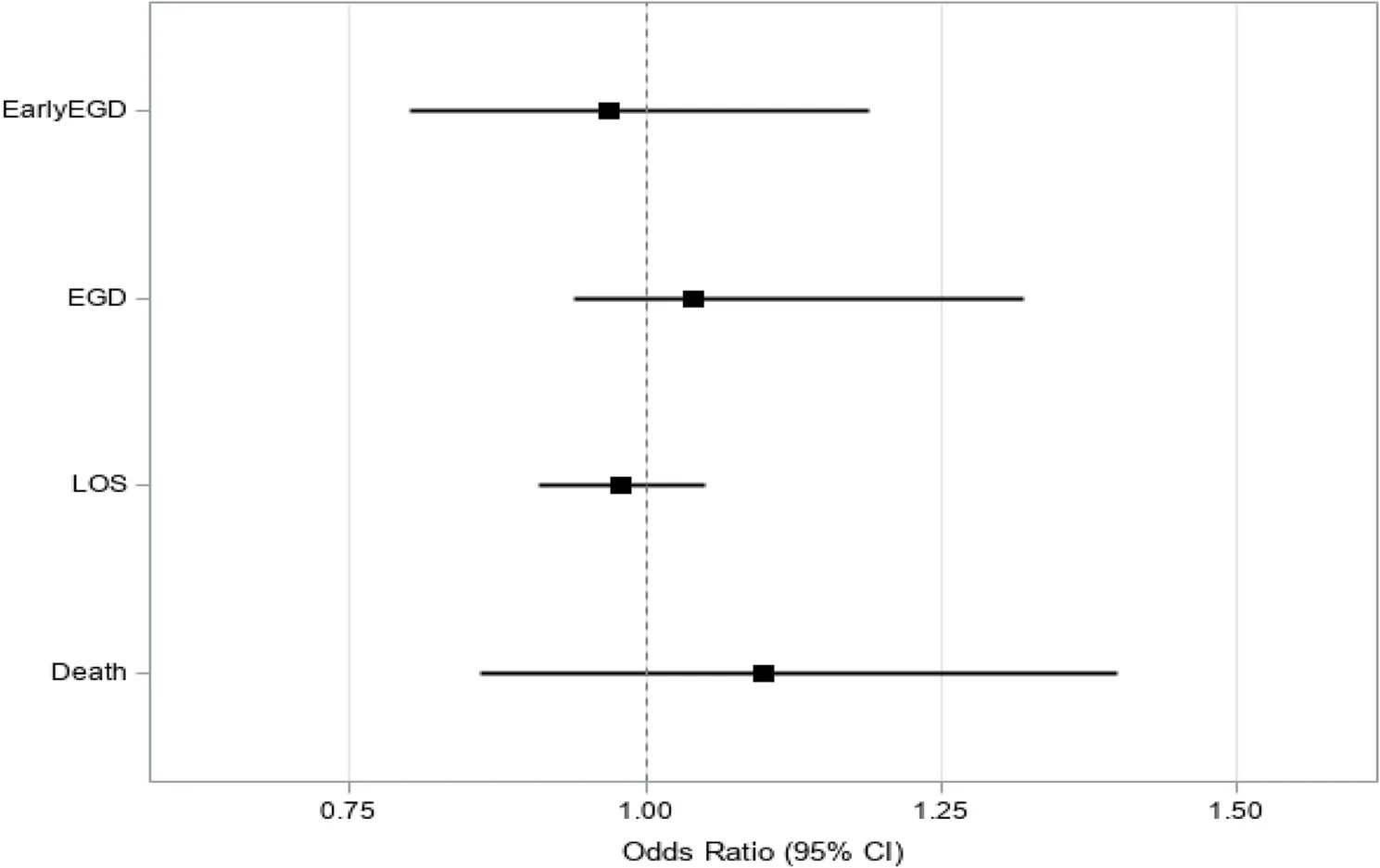
Forest Plot of Outcomes by July effect vs non-July effect. * LOS- Length of stay; EGD- Esophago-duodenoscopy *This plot represents adjusted odd ratios (aOR) distribution of outcomes; aOR greater than “1” shows a higher probability of individual outcome, while aOR less than ”1” lowers this probability. Statistical significance (*p* < 0.05) is when the confidence interval (CI) does not include “1’

Comprehensive multivariate models accounting for demographics, clinical comorbidities, hospital type, and insurance status are summarized in Tables 2, 3, 4 and 5. Figure 1 provides a visual distribution of adjusted odds ratios across outcomes. The consistency across subgroup analyses reinforces the central finding that resident turnover in July does not adversely affect mortality, procedure utilization and timing, or hospital stay among patients admitted with UGIB.

**Table 2:** Bivariate and multivariate logistic regression analysis of July effect on Mortality among patients with UGIB

Variables	Unadjusted Odds ratio (CI)	*p*-values	Adjusted odds ratio (CI)	*p*-values
July effect		0.7722		0.4406
Yes	1.03 (0.82–1.30)		1.10 (0.86–1.40)	
Age		0.0003		0.0740
18–34	0.41 (0.19–0.87)		0.59 (0.24–1.41)	
35–44	0.29 (0.15–0.59)		0.37 (0.17–0.80)	
45–54	0.71 (0.49–1.03)		0.96 (0.59–1.56)	
55–64	0.74 (0.57–0.97)		0.74 (0.50–1.10)	
=>65	Ref		Ref	
Race		0.3488		0.8616
Black	0.92 (0.66–1.25)		1.00 (0.73–1.39)	
Hispanic	0.72 (0.48–1.07)		0.87 (0.56–1.35)	
Asian/Pacific Islander	1.19 (0.64–2.19)		1.21 (0.61–2.41)	
White	Ref		Ref	
Sex		0.5993		0.8665
Male	1.06 (0.85–1.33)		0.98 (0.77–1.25)	
AKI		0.5913		0.3695
Yes	1.10 (0.78–1.54)		1.18 (0.82–1.70)	
Shock		0.3039		0.0133
Yes	0.48 (0.12–1.95)		0.16 (0.04–0.68)	
Cardiac arrest		< 0.0001		0.0314
Yes	28.58 (8.64–94.51)		11.32 (1.24-103.09)	
Resp.Failure		< 0.0001		0.0024
Yes	10.14 (7.48–13.74)		1.72 (1.21–2.43)	
Insurance		0.2907		0.6679
Medicaid	0.70 (0.48–1.04)		1.03 (0.62–1.69)	
Other payer	1.23 (0.64–2.37)		1.27 (0.58–2.77)	
Private	0.91 (0.67–1.25)		1.24 (0.85–1.83)	
Self-pay	0.68 (0.35–1.32)		1.59 (0.72–3.55)	
Medicare	Ref		Ref	

**Table 3:** Bivariate and Multivariate Logistic regression analysis of July effect on Length of Hospitalization (> 3days) among patients with UGIB

Variables	Unadjusted Odds ratio (CI)	*p*-values	Adjusted odds ratio (CI)	*p*-values
July effect		0.8203		0.5410
Yes	1.01 (0.94–1.09)		0.98 (0.91–1.05)	
Age		< 0.0001		< 0.0001
18–34	2.09 (1.74–2.50)		1.57 (1.22–2.03)	
35–44	1.81 (1.57–2.09)		1.63 (1.34–1.98)	
45–54	1.50 (1.33–1.68)		1.45 (1.23–1.70)	
55–64	1.19 (1.09–1.30)		1.23 (1.09–1.38)	
=>65	Ref		Ref	
Race		< 0.0001		< 0.0001
Black	0.86 (0.78–0.95)		0.81 (0.73–0.91)	
Hispanic	1.26 (1.11–1.43)		1.14 (0.99–1.30)	
Asian/Pacific Islander	1.09 (0.89–1.34)		1.15 (0.92–1.45)	
White	Ref		Ref	
Sex		0.4868		0.2954
Male	1.03 (0.95–1.11)		1.05 (0.96–1.14)	
AKI		0.2730		0.5128
Yes	0.94 (0.83–1.05)		1.04 (0.92–1.19)	
Shock		< 0.0001		0.0931
Yes	0.33 (0.29–0.49)		0.72 (0.49–1.06)	
Cardiac arrest		0.8563		0.1372
Yes	0.90 (0.27–2.95)		2.54 (0.74–8.69)	
Resp.Failure		< 0.0001		0.0427
Yes	0.36 (0.28–0.46)		1.34 (1.01–1.79)	
Insurance		< 0.0001		0.0380
Medicaid	1.37 (1.22–1.54)		1.03 (0.88–1.19)	
Other payer	1.23 (0.96–1.57)		1.06 (0.80–1.40)	
Private	1.46 (1.30–1.63)		1.14 (0.99–1.31)	
Self-pay	2.10 (1.72–2.57)		1.37 (1.10–1.70)	
Medicare	Ref		Ref	

**Table 4:** Bivariate and Multivariate Logistic regression analysis of July effect on EGD among patients with UGIB

Variables	Unadjusted Odds ratio (CI)	*p*-values	Adjusted odds ratio (CI)	*p*-values
July effect		0.6294		0.4412
Yes	1.03 (0.93–1.14)		1.04 (0.94–1.32)	
Age		< 0.0001		< 0.0001
18–34	0.40 (0.28–0.57)		0.52 (0.35–0.77)	
35–44	0.37 (0.27–0.51)		0.44 (0.31–0.62)	
45–54	0.48 (0.39–0.60)		0.54 (0.43–0.69)	
55–64	0.82 (0.71–0.94)		0.87 (0.74–1.03)	
=>65	Ref		Ref	
Race		0.0004		0.0927
Black	0.83 (0.72–0.96)		0.87 (0.75–1.01)	
Hispanic	0.71 (0.59–0.85)		0.82 (0.68–0.99)	
Asian/Pacific Islander	0.93 (0.63–1.29)		0.93 (0.68–1.28)	
White	Ref		Ref	
Sex		0.5848		0.9002
Male	0.97 (0.88–1.08)		1.01 (0.90–1.12)	
AKI		0.8326		0.1409
Yes	0.98 (0.84–1.15)		0.89 (0.75–1.04)	
Shock		0.0090		0.1639
Yes	1.64 (1.13–2.36)		1.32 (0.89–1.96)	
Cardiac arrest		0.3867		0.4248
Yes	1.80 (0.48–6.79)		1.77 (0.43–7.23)	
Respiratory Failure		0.5763		0.8207
Yes	1.09 (0.81–1.45)		0.96 (0.71–1.32)	
Insurance		< 0.0001		0.0005
Medicaid	0.52 (0.42–0.61)		0.75 (0.60–0.94)	
Other payer	0.63 (0.44–0.90)		0.77 (0.53–1.10)	
Private	0.86 (0.74–0.99)		1.13 (0.95–1.33)	
Self-pay	0.39 (0.27–0.58)		0.62 (0.41–0.93)	
Medicare	Ref		Ref	

**Table 5:** Bivariate and Multivariate Logistic regression analysis of July effect on Early EGD (< 24 h) among patients with UGIB

Variables	Unadjusted Odds ratio (CI)	*p*-values	Adjusted odds ratio (CI)	*p*-values
July effect		0.5730		0.7759
Yes	0.95 (0.978–1.15)		0.97 (0.80–1.19)	
Age		0.0240		0.3910
18–34	0.73 (0.31–1.71)		0.98 (0.38–2.42)	
35–44	0.71 (0.37–1.37)		0.87 (0.43–1.74)	
45–54	0.49 (0.30–0.81)		0.57 (0.32–0.99)	
55–64	0.77 (0.58–1.03)		0.86 (0.61–1.20)	
=>65	Ref		Ref	
Race		0.0865		0.0885
Black	1.34 (1.03–1.74)		1.39 (1.06–1.81)	
Hispanic	0.84 (0.56–1.24)		0.92 (0.61–1.39)	
Asian/Pacific Islander	0.84 (0.45–1.56)		0.85 (0.44–1.62)	
White	Ref		Ref	
Sex		0.0232		0.0370
Male	0.78 (0.63–0.97)		0.79 (0.64–0.99)	
AKI		0.8281		0.9110
Yes	1.04 (0.75–1.44)		0.98 (0.70–1.38)	
Shock		0.2004		0.1177
Yes	0.56 (0.24–1.36)		0.49 (0.20–1.20)	
Cardiac arrest		0.7726		0.9283
Yes	1.43 (0.13–15.83)		1.11 (0.11–11.52)	
Respiratory Failure		0.1863		0.1185
Yes	0.66 (0.36–1.22)		0.59 (0.30–1.15)	
Insurance		0.0218		0.5091
Medicaid	0.59 (0.38–0.90)		0.68 (0.41–1.12)	
Other payer	0.53 (0.22–1.30)		0.61 (0.25–1.51)	
Private	0.73 (0.53–1.02)		0.88 (0.60–1.30)	
Self-pay	0.60 (0.28–1.28)		0.79 (0.35–1.78)	
Medicare	Ref		Ref	

## Discussion

The “July phenomenon,” commonly referred to as the “July effect,” posits that the annual influx of new medical residents and fellows in teaching hospitals during the summer months may negatively impact patient outcomes. This concern stems from the notion that new residents, often lacking practical experience and familiarity with institutional workflows, may inadvertently contribute to increased rates of complications, prolonged hospital stays, and higher mortality [12]. This study sought to assess whether such a phenomenon affects clinical outcomes in patients admitted with acute upper gastrointestinal bleeding (UGIB), a medical emergency requiring prompt evaluation and often endoscopic intervention, and one that carries a substantial mortality risk.

One of the study’s most striking findings is the absence of a significant difference in mortality rates between the July effect group and the non-July effect group. With an overall mortality rate of approximately 3% among the sample population with UGIB, the adjusted odds ratios (aOR) for mortality between the July and non-July effect groups is (1.10 [0.86–1.40] *p* = 0.4406) statistically non-significant. This finding suggests that the turnover of medical residents in July does not contribute to the risk of death in patients with UGIB. This contrasts with some previous studies that have linked the July effect with worse patient outcomes, including higher mortality rates, particularly in complex or high-risk scenarios [10]. From our study we believe that the intake of new residents and fellows at the start of the session in July is more of a coincidence than a cause of mortality outcomes among patients with UGIB.

Similarly, there is no difference in rate of endoscopy between the two groups with UGIB (1.04 [0.94–1.32], *p* = 0.4412) and Early EGD (0.97 [0.80–1.19], *p* = 0.7759). This indicates that the turnover of medical trainees in July did not delay access to endoscopic treatment or timing of the procedure. In fact, the timely administration of endoscopy may be more dependent on hospital protocols, availability of endoscopist or endoscopy units, staffing levels, and the severity of the cases rather than the month of the year. This result challenges the notion that the intake of new residents leads to significant delays in critical care interventions.

The length of hospitalization between the two groups (July vs. non-July effect) was not significantly different (0.98 [0.91–1.05], *p* = 0.5410). It is surprising that despite the inexperience of new residents who might require more supervision or order more diagnostic tests, consultations, or longer procedures to ensure patient safety, these do not influence patients’ stay.

### Limitation of the study

The NIS dataset is robust, covering a wide variety of hospitals across the United States, it is not comprehensive. With less than 20% of all hospital discharges accounted for, the absence of a clear July effect in this study should not be considered definitive. In addition, the NIS lacks detailed clinical information on the severity of upper gastrointestinal bleeding, including endoscopic findings, laboratory values, transfusion requirements, and timing of clinical interventions. The inability to adjust for UGIB severity may have introduced residual confounding and could have influenced the observed associations and their interpretation. Variations in hospital policies, regional practices, possible patient misclassification and patient demographics across different institutions may influence the generalizability of the findings. Future research, potentially using more granular hospital-level data, could provide further insights into whether certain types of hospitals or specific patient populations are more affected by the July turnover.

## Conclusions

This study provides evidence that counters the notion of the July effect as a detrimental influence on patient outcomes for UGIB. Rather, the findings support the reliability of healthcare delivery in teaching hospitals, even during periods of resident turnover. These results underscore the importance of continued support and investment in graduate medical education (GME) programs, which ensure a steady pipeline of well-trained clinicians without compromising patient care quality. We advocate for ongoing quality assurance and structured supervision strategies to sustain patient-centered care year-round.

### ICD CODES

**Table:** 

ICD-10-CM diagnosis and Procedures	ICD- 10- codes
UGIB (non-variceal)	K270, K280, K2101, K2211, K2901, K2921, K2961, K2971, K2981, K2991, K250, K260, K922, K31811,
Shock	R571
Cardiac arrest	I468, I469
Acute Kidney Injury	N170, N171, N172, N178, N179
Respiratory failure	J9601, J9602
Therapeutic EGD	0W3P7ZZ, 0W3P8ZZ

## Data Availability

Data for this study was obtained from the Healthcare Cost and Utilization Project (HCUP), sponsored by the Agency for Healthcare Research and Quality. The authors complied with the HCUP Data Use Agreement and all associated data security and confidentiality requirements. The NIS data for the period of study (2016-2020) can be accessed at: https://www.hcup-us.ahrq.gov [13].

## Abbreviations

UGIB: Upper gastrointestinal bleeding
NVUGIB: Nonvariceal upper gastrointestinal bleeding
EGD: Esophagogastroduodenoscopy
GME: Graduate medical education
AKI: Acute Kidney Injury
NIS: National Inpatient Sample
LOS: Length of stay
GAVE: Gastric antral vascular ectasia
HCUP: Healthcare Cost and Utilization Project
AHRQ: Agency for Healthcare Research and Quality
aOR: adjusted odds ratio
ACG: American College of Gastroenterology

## References

[1] Buchwald D, Komaroff AL, Cook EF, Epstein AM. Indirect costs for medical education. Is there a July phenomenon? Arch Intern Med. 1989;149(4):765–8. 2495778

[2] Zogg CK, Metcalfe D, Sokas CM, Dalton MK, Hirji SA, Davis KA, et al. Reassessing the July Effect: 30 Years of Evidence Show No Difference in Outcomes. Ann Surg. 2023;277(1):E204–11. 33914485 10.1097/SLA.0000000000004805 PMC8384940

[3] Blough JT, Jordan SW, De Oliveira GS, Vu MM, Kim JYS. Demystifying the July Effect in plastic surgery: A multi-institutional study. Aesthetic Surg J. 2018;38(2):212–24. 10.1093/asj/sjx099 29040397

[4] Lau JYW, Yu Y, Tang RSY, Chan HCH, Yip HC, Chan SM, et al. Timing of Endoscopy for Acute Upper Gastrointestinal Bleeding. N Engl J Med. 2020;382(14):1299–308. 32242355 10.1056/NEJMoa1912484

[5] Alali AA, Barkun AN. An update on the management of non-variceal upper gastrointestinal bleeding. Gastroenterol Rep. 2023;11. 10.1093/gastro/goad011 PMC10027415 36949934

[6] Bai L, Jiang W, Cheng R, Dang Y, Min L, Zhang S. Does Early Endoscopy Affect the Clinical Outcomes of Patients with Acute Nonvariceal Upper Gastrointestinal Bleeding? A Systematic Review and Meta-Analysis. Gut Liver. 2023;17(4):566–80. 36578195 10.5009/gnl220291 PMC10352052

[7] Spiegel BMR, Vakil NB, Ofman JJ. Endoscopy for Acute Nonvariceal Upper Gastrointestinal Tract Hemorrhage: Is Sooner Better? A Systematic Review [Internet]. Available from: http://archinte.jamanetwork.com/10.1001/archinte.161.11.1393 11386888

[8] Abougergi MS, Travis AC, Saltzman JR. Impact of day of admission on mortality and other outcomes in upper GI hemorrhage: A nationwide analysis. Gastrointest Endosc. 2014;80(2). 10.1016/j.gie.2014.01.043 24674354

[9] Gangu K, Basida S, Awan RU, Butt MA, Reed A, Afzal R, et al. July effect in clinical outcomes of esophagogastroduodenoscopy performed at teaching hospitals in the United States. Bayl Univ Med Cent Proc. 2023;36(4):478–82. 10.1080/08998280.2023.2204804 PMC10269412 37334097

[10] Phillips DP, Barker GEC. A July spike in fatal medication errors: A possible effect of new medical residents. J Gen Intern Med. 2010;25(8):774–9. 20512532 10.1007/s11606-010-1356-3 PMC2896592

[11] Asotibe JC, Shaka H, Akuna E, Shekar N, Shah H, Ramirez M, et al. Outcomes of Non-Variceal Upper Gastrointestinal Bleed Stratified by Hospital Teaching Status: Insights From the National Inpatient Sample. Gastroenterol Res. 2021;14(5):268–74. 10.14740/gr1437 PMC8577599 34804270

[12] Barry WA, Rosenthal GE. Is there a July phenomenon? The effect of July admission on intensive care mortality and length of stay in teaching hospitals. J Gen Intern Med. 2003;18(8):639–45. 10.1046/j.1525-1497.2003.20605.x . PMID: 12911646; PMCID: PMC1494902. 12911646 10.1046/j.1525-1497.2003.20605.x PMC1494902

[13] Healthcare Cost and Utilization Project (HCUP). HCUP NIS Database Documentation. Agency for Healthcare Research and Quality. Published June 2024. Available from: www.hcup-us.ahrq.gov/db/nation/nis/nisdbdocumentation.jsp

